# Some Points on Bridgework

**Published:** 1894-10

**Authors:** H. J. Ray


ARTICLE VI.

### SOME POINTS ON BRIDGEWORK.

BY DR. H. J. RAY.

Read before the South Carolina State Dental Association, May,'94.

As briefly as possible I wish to present some points on bridge-work. You are all doubtless posted on the methods now practiced, and will readily appreciate the new features in the following case: To illustrate, we will take a typical case, say from the second lower bicuspid to the third molar. First, take an impression in Teagues Impression compound, and into this pour directly a metal die and articulate. Next, make a gold crown for either tooth. In this case we will crown the bicuspid. I crown but one of my abutments, so in the molar we will prepare as large a cavity as the living pulp will allow on the anterior surface, from the grinding surface to the gum margin. This cavity is filled with the hardest cohesive gold foil, into this and filling a depression is drilled to accomodate backend of the the bridge, which ends in a gold bar which rests looselyin this depression.

Instead of using a *bar* from the crown to the molar to attach the teeth to, a wide piece of gold plate is used with the flat side next the alveolar ridge. This can be swagged down on the die to fit the ridge close, or bent upward ovally, leaving a slight space between the gold and the ridge. A sufficiently strong bar is soldered on to the molar end of this to fit into the molar cavity, as already described.

Now, the next thing is attaching the teeth. These are not soldered to the bridge at all, but are cemented into gold sockets, which are soldered on to this piece of flat gold plate. I use Genese's pinless teeth and measure gold bands to fit around them, solder the ends of this band together and press the teeth into them to shape them, and then wax the bands in position on the bridge of the investment, remove the wax and solder them to the bridge, making the solder to flow freely between the band, uniting them all solidly, thus making the piece very strong. These sockets are from one-eighth to one-sixth of an inch deep. Place the piece now on the die articulation and grind the porcelain teeth to the proper occlusion, cement them into their sockets, and the bridge is ready to be placed in the mouth in the usual manner.

I make my die in two pieces—first pour my die proper and let it cool, then pour on top of it either the same metal or lead, about one-fourth of an inch more. These two parts separate and fit into each other accurately; and when I make the articulation I use both parts, only the base being in the plaster. I can then lift my die proper off its base and work on it as I like in swagging or filing, and fit it back into its base for trial or articulation. If the crown is made stiff to fit accurately, and all the parts strong throughout, it will be held securely in position and be strong enough for all practical purposes.

This process insures an accurate fit as only one tooth is covered— and that the most suitable one in the case—necessitates grinding only one tooth, allows a living pulp to remain in either tooth, permits the natural motion of the teeth, avoids heating the porcelain teeth, so that there is no danger of fracturing them, presents a natural and artistic appearance, and makes it practically impossible to break a tooth from the bridge, and should it happen, allows it to be replaced very easily without removing the bridge.—*Southern Dental Journal.*

